# 25(OH)Vitamin D Deficiency and Calcifediol Treatment in Pediatrics

**DOI:** 10.3390/nu14091854

**Published:** 2022-04-29

**Authors:** Luis Castano, Leire Madariaga, Gema Grau, Alejandro García-Castaño

**Affiliations:** Cruces University Hospital, Biocruces Bizkaia Health Research Institute, UPV/EHU, CIBERER/CIBERDEM, Endo-ERN, 48903 Barakaldo, Spain; leyre.madariagadominguez@osakidetza.eus (L.M.); mariagema.graubolado@osakidetza.eus (G.G.); alejandro.garciacastano@osakidetza.eus (A.G.-C.)

**Keywords:** nutritional rickets, genetic forms of rickets, vitamin D deficiency, vitamin D treatment, calcifediol

## Abstract

Vitamin D is essential for the normal mineralization of bones during childhood. Although diet and adequate sun exposure should provide enough of this nutrient, there is a high prevalence of vitamin D deficiency rickets worldwide. Children with certain conditions that lead to decreased vitamin D production and/or absorption are at the greatest risk of nutritional rickets. In addition, several rare genetic alterations are also associated with severe forms of vitamin-D-resistant or -dependent rickets. Although vitamin D3 is the threshold nutrient for the vitamin D endocrine system (VDES), direct measurement of circulating vitamin D3 itself is not a good marker of the nutritional status of the system. Calcifediol (or 25(OH)D) serum levels are used to assess VDES status. While there is no clear consensus among the different scientific associations on calcifediol status, many clinical trials have demonstrated the benefit of ensuring normal 25(OH)D serum levels and calcium intake for the prevention or treatment of nutritional rickets in childhood. Therefore, during the first year of life, infants should receive vitamin D treatment with at least 400 IU/day. In addition, a diet should ensure a normal calcium intake. Healthy lifestyle habits to prevent vitamin D deficiency should be encouraged during childhood. In children who develop clinical signs of rickets, adequate treatment with vitamin D and calcium should be guaranteed. Children with additional risk factors for 25(OH)D deficiency and nutritional rickets should be assessed periodically and treated promptly to prevent further bone damage.

## 1. Introduction

The vitamin D endocrine system (VDES) plays a pivotal role in maintaining phosphorus and calcium homeostasis and is essential for bone health. In addition, it is involved in several physiological processes related to cell growth and differentiation, immune response, and cardiovascular function [1,2]. Deficiency in children is the main cause of rickets worldwide [3].

Although the clinical picture of rickets had already been described in several medical books in the 17th century, a Polish doctor—Sniadecki—was the first to relate rickets to the lack of sun exposure. In 1932, Windaus described the biochemical structure of vitamin D2 (ergocalciferol) and vitamin D3 (cholecalciferol). Finally, in subsequent years, Jeans and Stearns described how children with rickets improved after treatment with these vitamins [3,4].

Even though vitamin D3 is the threshold nutrient of the VDES, the direct measurement of circulating vitamin D3 itself is not a good marker of the nutritional status of the system. Calcifediol, or 25(OH)D, serum levels are used to assess VDES status. At the beginning of the 21st century, several studies identified the levels of 25(OH)D necessary to optimize calcium absorption, normalize parathyroid hormone (PTH)serum levels, and optimize bone mineralization. The measurement of total circulating 25(OH)D concentration, due to its long half-life and high concentration, constitutes a robust and reliable biomarker of the nutritional status of the VDES. This measurement is used by health authorities and scientific societies in America and Europe to establish normality status, the definition of VDES deficiency and degrees of VDES insufficiency, on which to establish dietary reference intake, as well as population monitoring of VDES status, despite the lack of consensus between the different scientific associations involved (Table 1) [5]. However, several studies have shown population differences in 25(OH)D levels, and this should be taken into account, as universally optimal levels of vitamin D are difficult to define [6].

Several studies in adults have described the association of vitamin D deficiency with other pathologies not related to bone metabolism, namely cancer, cardiovascular disease, autoimmune diseases, allergies, and infectious diseases [7]. On the other hand, although elevated calcifediol levels have also been associated with an increased mortality rate due to an increased risk of cancer or cardiovascular disease [8,9], recent large-scale intervention studies did not observe an increased mortality risk, contradicting observational data [10].

The aim of this narrative review is to describe the importance of VDES in childhood, the risk factors of vitamin D deficiency, and the associated clinical picture, specifically in the pediatric population, in addition to the different prevention and treatment schedules proposed. In addition, rickets of genetic origin and their treatment is discussed. Articles were retrieved by search in PubMed up to January 2022, focusing on the most relevant studies.

## 2. Metabolism of the Vitamin D Endocrine System: Role of Genetic Factors

**Figure 1:**
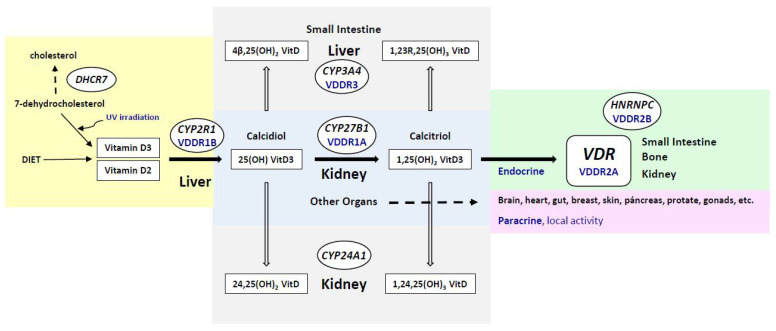
Schematic representation of VDES metabolism and the main genes involved. The *CYP2R1*, *CYP27B1*, *CYP24A1*, and *CYP3A4* genes encode the enzymes vitamin D 25-hydroxylase, 25-hydroxyvitamin D3-1-alpha-hydroxylase, vitamin D 24-hydroxylase and cytochrome P450 3A4, respectively. The *VDR* gene encodes the vitamin D receptor. The *DHCR7* gene encodes delta-7-dehydrocholesterol reductase. The *HNRNPC* gene encodes Heterogeneous Nuclear Ribonucleoprotein C. VDDR1A, vitamin D-dependent rickets type 1A; VDDR1B, vitamin D hydroxylation-deficient rickets type 1B; VDDR2A, vitamin D-dependent rickets type 2A; VDDR2B, vitamin D-dependent rickets type 2B with normal vitamin D receptor; VDDR3, dominant vitamin D-dependent rickets type 3.

Vitamin D3 is produced in the skin from 7-dehydrocholesterol by UV (ultraviolet) irradiation (whereas vitamin D2 is derived from the plant sterol ergosterol). Vitamin D metabolites are carried in the blood and are bound to vitamin D binding protein (DBP) and albumin (Figure 1). Hydroxylation takes place, principally in the liver, producing calcifediol (25 hydroxyvitamin D3; 25(OH)D_3_), the major circulating form of VDES. This hydroxylation of both forms of vitamin D, vitamin D2, and vitamin D3, is carried out by the enzyme vitamin D, 25-hydroxylase (encoded by the *CYP2R1* gene). A second hydroxylation to synthesize calcitriol (or 1,25 dihydroxyvitamin D;1,25(OH)2D) is catalyzed by the enzyme 25-hydroxyvitamin D3-1-alpha-hydroxylase (encoded by the *CYP27B1* gene); this occurs mainly in the kidneys for its endocrine actions, and in cells of multiple tissues, organs, and systems (e.g., skin, parathyroid gland, breast, colon, prostate, and lung), as well as in cells of the immune system and bone, for its auto/paracrine actions (Figure 1). Calcitriol mediates most VDES hormonal actions by stimulating the vitamin D receptor (VDR). In addition, another important metabolite, 24,25(OH)2 vitamin D, is produced by the enzyme vitamin D 24-hydroxylase (encoded by the *CYP24A1* gene); this process initiates the catabolic process and the production of calcitroic acid. Furthermore, cytochrome P450 3A4 (encoded by the *CYP3A4* gene) catalyses the inactivation of the active hormone 1,25(OH)2D, principally in the liver.

Regarding the involvement of heritability in vitamin D status, several twin and family studies have reported it to be between 20% and 85% [5,6]. Thus, several single-nucleotide polymorphisms (SNPs) and mutations in genes closely related to vitamin D3 synthesis, activation, and degradation (Figure 1) have been associated with different serum levels of calcifediol.

Variants in the *CYP2R1* gene may result in calcifediol deficiency [11]. Vitamin D hydroxylation-deficient rickets type 1B (VDDR1B; MIM 600081) is caused by homozygous, compound heterozygous, or heterozygous mutations in the *CYP2R1* gene, with a more severe disease phenotype with homozygosity and a less severe phenotype with heterozygosity [12].

Some *CYP27B1* variants are related to vitamin D status and have been found to be associated with lower serum levels of calcifediol [13]. Homozygous and compound heterozygous mutations in the *CYP27B1* gene cause vitamin D-dependent rickets type 1A (VDDR1A; MIM 264700), a defect in the synthesis of the active form of vitamin D.

The *VDR* gene encodes the vitamin D receptor, which mediates the action of the active form of vitamin D on cells. VDR-RXR (retinoid X receptors) heterodimers bind to specific response elements on DNA and activate the transcription of calcitriol-responsive target genes. Vitamin D-dependent rickets type 2A (VDDR2A; MIM 277440) is caused by mutations in the *VDR* gene.

Vitamin D-dependent rickets type 2B with normal VDR (VDDR2B; MIM 600785) is a rare form of rickets due to an abnormal protein (HNRNPC) that interferes with the function of the *VDR* gene [14].

Dominant vitamin D-dependent rickets type 3 (VDDR3; MIM 619073) is caused by accelerated inactivation of the VDES metabolites, 25(OH)D_3_ and 1,25(OH)2D, due to gain-of-function mutations in the *CYP3A4* gene [15].

Moreover, other genes have been shown to be involved in VDES regulation. The group-specific component (*GC*) gene encodes the vitamin DBP, and some SNPs in this gene have been associated with lower serum 25(OH)D levels [16]. Furthermore, *CYP24A1* catalyzes the inactivation of both the calcidiol prohormone (and is a cornerstone of the VDES) and calcitriol, the active hormonal form. Finally, the *DHCR7* gene that encodes the delta-7-dehydrocholesterol reductase catalyzes the final step of cholesterol biosynthesis [17].

Evidence about the involvement of other gene variations in vitamin D status, namely retinoid X receptors (*RXR*), calcium-sensing receptors (*CASR*), *NPY*, *FOXA2*, *SSTR4*, *FGF23*, *CUBN*, *LRP2*, and *IVL*, is limited [18,19].

Genome-wide association studies have confirmed strong associations with calcifediol at four loci, *GC* (index SNP: rs2282679), *DHCR7/NADSYN1* (rs12785878), *CYP2R1* (rs10741657), and *CYP24A1* (rs17216707). In addition to these four known loci, two novel loci, *SEC23A*, Sec23 homolog A, coat protein complex II component (rs8018720) and *AMDHD1*, amidohydrolase domain containing 1 (rs10745742), which are genes outside of the VDES metabolism pathway, have been reported to be involved in serum calcifediol homeostasis [20].

## 3. Prevalence of Calcifediol Deficiency and Insufficiency in Childhood

During the 20th century, the general recommendations of controlled sun exposure and fortification of milk with vitamin D were designed to eradicate rickets, but in the last few years, many studies have reported an increase in the incidence of rickets worldwide. The reported prevalence is variable, depending on the population analyzed and on the reference levels used for the definition of calcifediol deficiency and insufficiency [5,7].

In children, various observational studies have highlighted the need for 25(OH)D serum levels over 50 nmol/L to prevent rickets and over 75 nmol/L from improving other aspects related to health. However, the cut-off levels used for the definition of calcifediol deficiency and insufficiency are still controversial [3]. The most recent global consensus recommendations for the management of nutritional rickets determine sufficiency as 25(OH)D levels between 50 and 250 nmol/L (20–100 ng/mL), insufficiency from 30 to 50 nmol/L (12–20 ng/mL), and deficiency with 25(OH)D levels below 30 nmol/L (12 ng/mL). These cut-off points for the different definitions are based on the observation that children with 25(OH)D serum levels lower than 50 nmol/L (20 ng/mL) already have elevated serum alkaline phosphatase levels, and those below 40–45 nmol/L (16–18 ng/mL) also show radiological signs of rickets [21].

Calcifediol deficiency is higher in Asia than in other continents [7]. In developing countries, infants are the most affected population, with the highest risk for developing vitamin D deficiency, especially those that are exclusively breastfed. However, recently in Europe, the risk of vitamin D deficiency has increased the most in adolescents. In 2014, a population study in England reported that the prevalence of vitamin D deficiency among boys aged 4 to 10 years was 20%, and it was even higher (24%) in girls aged 11 to 18 years [22]. Other studies evaluating the prevalence of calcifediol deficiency in developed countries highlight these high rates of low 25(OH)D serum levels in adolescents. In a recent report on the general pediatric population from the North of Spain (291 healthy children, with a median age of 9 years), severe calcifediol deficiency (25(OH)D < 25 nmol/L) was found in 1.4% [23]. However, this prevalence increased to 18% if the 25(OH)D threshold was <50 nmol/L, and only 44% of the children were considered to have sufficient calcifediol (25(OH)D > 75 nmol/L), according to the American Institute of Medicine. This study showed a relationship between low calcifediol serum levels and non-Caucasian ethnic groups, months with a low intensity of sunlight (end of autumn, winter, and beginning of spring), and weight [23]. Similarly, in the United States, reports have shown a prevalence of insufficient levels of calcifediol in 15% of the pediatric population and severe deficiency (<25 nmol/L or 10 ng/mL) in 1–2% [24,25,26].

Furthermore, studies in different populations with a higher risk of developing calcifediol deficiency, such as populations from higher latitudes or with darker skin, have shown different adaptive mechanisms that favor a more efficient VDES metabolism [27].

## 4. Risk Factors for Calcifediol Deficiency in Childhood

### 4.1. Lack of Sun Exposure

The synthesis of vitamin D in the skin depends on the degree of exposure to sunlight, specifically ultraviolet-B (UV-B) rays. In people with light skin pigmentation, sufficient skin synthesis of vitamin D can be achieved through 10 to 15 min of sun exposure between 10 am to 3 pm during spring, summer, and autumn (necessary exposure area: arms and legs; or hands, arms, and face). Most people with medium skin pigmentation, such as children of south Asian ancestry, require three times more sun exposure than people with light skin pigmentation to achieve the same serum levels of 25(OH)D. Finally, people with dark or very dark skin pigmentation, such as children of African ancestry, require 6 to 10 times higher sun exposure to achieve the same levels of calcifediol [28,29].

The dose of UV radiation necessary to produce 1000 IU of vitamin D that will guarantee sufficient 25(OH)D serum levels is achieved with 25% of the minimal erythema dose. This is equivalent to 10 to 15 min of sun exposure to 25% of the body surface area (face, arms, hands) without sunscreen and during the central hours of the day. Regarding sun exposure, people living beyond 40° north of the equator, the presence of dark skin, the excessive use of very high SPF sunscreen, the limitation of outdoor activities, and dress habits in which the skin is not shown are risk factors for vitamin D deficiency [23]. In addition, immigrant/refugee children who move to countries at higher latitudes may have many of the risk factors listed above and are, therefore, very susceptible to health issues related to vitamin D deficiency.

### 4.2. Pregnancy and Lactation

Serum levels of 1,25(OH)2D increase early in pregnancy regardless of PTH levels and will remain elevated until delivery. This means that pregnant women increase their 25(OH)D requirements. The physiological explanation for this fact is probably the need to maintain adequate maternal-fetal homeostasis in relation to calcium. A recent epidemiological study with pregnant women and their newborns from Spain shows a high rate of insufficiency (64% of pregnant mothers and 44% of newborns with 25(OH)D levels <50 nmol/L) and highlighted that, during low sun-exposure periods, 25(OH)D serum levels were higher in the newborn than in the mother. This could be understood as a protective physiological mechanism to ensure calcium homeostasis in the newborn [30]. Once pregnancy is concluded, serum levels of 1,25(OH)2D return to preconception ranges, but the mother who breastfeeds the baby will continue to have high calcifediol needs. In conclusion, pregnancy and breastfeeding are critical periods of high vitamin D needs. In addition, in exclusively breastfed infants, a minimum daily vitamin D intake level of 400 IU should be ensured, as the content in breast milk is low (15–50 IU/L [0.4–1.2 µg/L]) even in mothers with normal 25(OH)D serum levels [31].

Calcifediol deficiency during pregnancy has been associated with an increased prevalence of preeclampsia, low birth weight, and neonatal hypocalcemia [32].

### 4.3. Prematurity

Premature infants are at a higher risk of developing rickets due to calcium and phosphorus deficiency and to lower concentrations of 25(OH)D. In addition, the premature newborn has less time to accumulate vitamin D from the mother as the pregnancy period is shorter. The third trimester of gestation is a critical time for bone health since it is when most of the fetal skeleton is calcified, and this requires greater activation of 25(OH)D to 1,25(OH)2D from the maternal kidneys and placenta. Therefore, calcifediol deficiency in the mother during this important period, or premature birth, can cause calcifediol deficiency in the fetus and, in severe cases, fetal rickets [33,34,35].

### 4.4. Obesity

The prevalence of calcifediol deficiency in overweight and obese children, a situation that is increasing in Western societies, is very high (36–93%). In accordance with this, excessive fat accumulation in children and calcifediol deficiency frequently appear in the association. Vitamin D deficiency is involved in the inflammatory processes that occur in obese children and affects insulin secretion and resistance. Several hypotheses have tried to explain the association between low serum 25(OH)D levels and obesity, and the most widely accepted is that adipose tissue traps fat-soluble vitamin D. In addition, the frequently sedentary lifestyle of the obese is associated with less sun exposure, would contribute as a risk factor. Moreover, hepatic steatosis present in many obese children could be responsible for decreasing the hepatic synthesis of 25(OH)D [3,36].

### 4.5. Children with Chronic Diseases

○ Kidney disease: calcifediol deficiency is frequent and may be severe in children and adults with chronic kidney disease [37].
○ Liver disease: severe chronic liver disease leads to altered synthesis and impaired absorption of vitamin D due to impaired bile acid production or intestinal edema secondary to portal hypertension [36].
○ Inflammatory bowel disease: vitamin D deficiency is very frequent in children with inflammatory bowel disease and other malabsorption bowel diseases such as cystic fibrosis due to malabsorption. In addition, several studies have reported a higher severity of inflammatory bowel disease secondary to vitamin D deficiency [38].

### 4.6. Others

○ Drugs: some treatments may interfere with vitamin D metabolism, either by increased catabolism of 25(OH)D or 1,25(OH)2D (antiepileptic or antiretroviral drugs), inhibition of intestinal absorption of vitamin D (glucocorticoids), or an increased requirement due to hydroxylation blocking (ketoconazole) [39].
○ Nephrotic syndrome: low serum levels of 25(OH)D can be seen in relation to an increased urinary loss of vitamin D-binding protein and of vitamin D itself [40].

## 5. Clinical Manifestations of Calcifediol Deficiency in Childhood

### 5.1. Calcifediol Deficiency and Bone Metabolism in Childhood

The main role of the VDES is to maintain adequate serum levels of calcium and phosphorus in order to ensure normal bone mineralization (ossification of the preosseous cartilage requires sufficient deposits of calcium and phosphorus to form the hydroxyapatite crystals). This is achieved by three different mechanisms: the stimulation of intestinal calcium absorption, the increase of bone resorption in the presence of hypocalcemia, and the increase of renal calcium reabsorption when necessary. In addition, in the child, 1,25(OH)2D has an active role in the development of the growth plate.

The various situations that may lead to a deficiency and/or inadequate function of the VDES produce a decrease in the absorption of calcium and phosphorus at the intestinal level and, therefore, associated hypocalcemia. This, in turn, generates compensatory hyperparathyroidism, which increases phosphaturia and produces bone demineralization or osteomalacia. In growing children, the epiphyseal plate will also be affected, leading to calcium-deficiency rickets.

**Figure 2:**
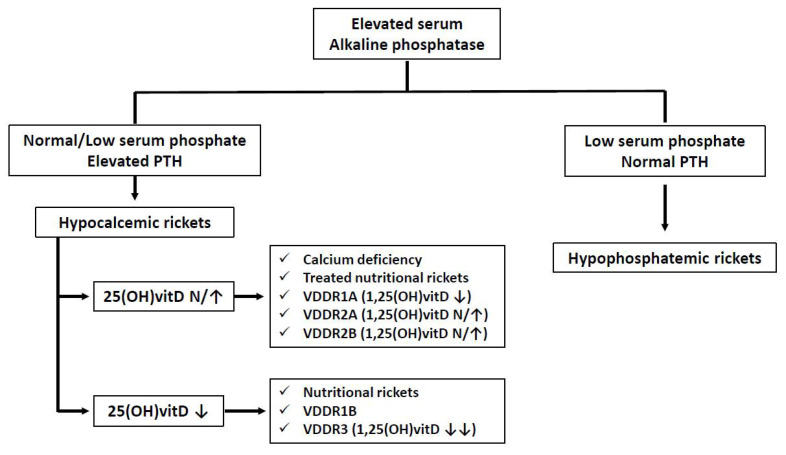
Diagnostic algorithm of the different forms of rickets.

Calcium-deficiency rickets can be caused by a shortage of calcium intake, vitamin D deficiency with an associated decrease of intestinal calcium absorption (known as nutritional rickets), or a failure in the normal activation of vitamin D. Rickets of genetic origin due to defects in the metabolism of vitamin D is rare but should be part of the differential diagnosis of children with clinical and radiological signs of rickets (Figure 2).

Characteristically, the most affected bones in nutritional rickets will be those with more active growth, especially in the first years of life. In the epiphyseal plate, the increase in osteoid bone tissue and the hypertrophy and proliferation of the growth plate produces the typical widening of the epiphysis. Other typical radiological findings are osteopenia with cortical thinning, Looser–Milkman striae, frayed metaphyses, small and irregular epiphyseal ossification centers, and delayed appearance of ossification centers.

Clinical manifestations include the development of rachitic rosary (palpable nodulations in the costochondral junctions), cranial deformities in infants (craniotabes), thoracic alterations (pectus carinatum), deformities of the lower extremities in children who have started walking (genu varum or genu valgum and leg bowing), dental anomalies (delayed tooth eruption and enamel defects), and increased fracture risk.

Apart from these anomalies in bones and cartilages, other manifestations resulting from hypocalcemia and muscle and ligament involvement are also described in rickets: respiratory complications such as recurrent bronchopneumonia and laryngospasm, neuromuscular alterations such as hypotonia and seizures, muscular weakness or pain, and cardiac anomalies such as arrhythmias and cardiomyopathy. The severity of the clinical manifestations and the biochemical and hormonal anomalies will depend on the gravity and duration of the disease (Table 2).

In those children for whom growth has ended, we will not find the characteristic radiological alterations of deficiency rickets, but the development of osteomalacia will produce a significant deterioration of adult bone health, especially in women, as bone mass is mostly generated at this stage of life (90% of adult bone mass is present by the end of adolescence) [5,21].

### 5.2. Other Anomalies Associated with Calcifediol Deficiency

In children, as in adults, the VDES has other effects on health beyond calcium homeostasis and bone development. Although some epidemiological studies have suggested an association between calcifediol deficiency and multiple pathologies outside bone anomalies, a clear causal relationship has not been established to date. Moreover, low levels of 25(OH)D have been associated with hypertension, hyperglycemia, metabolic syndrome, respiratory infections, asthma, and food allergies in adolescents in the United States, although intervention studies carried out on these pathologies have not shown any preventive effect with vitamin D treatment. In addition, various infectious diseases with a high incidence in childhood (otitis media, urinary tract infection, pneumonia, influenza, and other acute respiratory infections) have also been associated with calcifediol deficiency, but again, randomized controlled trials using vitamin D have failed to report conclusive data about its utility in the prevention of these diseases [3,28].

## 6. Treatment of Calcifediol Deficiency in Childhood

### 6.1. Vitamin D for the Prevention of Nutritional Rickets

The amount of vitamin D in a regular diet is small, as most foods naturally contain limited quantities of this element. Only fatty fish, such as cod or salmon, contain higher amounts, but these are not usually consumed in large quantities by children. For infants under one year of age, breast milk contains insufficient quantities of vitamin D and depends on the vitamin D status of the mother, while formula milk is usually fortified to contain about 400 IU/L of vitamin D. However, as infants decrease their milk intake when they start food diversification, the intake of vitamin D soon becomes insufficient.

Over time, the recommended dietary intake of vitamin D has changed according to the blood threshold levels of vitamin D considered normal. The fundamentals of vitamin D treatment are the prevention of nutritional rickets in infancy [41,42]. However, as other roles apart from optimizing bone mineralization have been attributed to this micronutrient, such as the modulation of the immune system and, therefore, the prevention of infections, treatment with vitamin D has also been used to prevent infections, especially respiratory infections and asthma crises [43,44]. Other goals of global vitamin D treatment are the optimization of growth and prevention of stunting in children [45].

The recommended dose of vitamin D as a prophylactic treatment in the first year of life is 400 IU/day. Higher doses up to 1600 IU/day have been used, with little or no additional benefits in preventing nutritional rickets and side effects appearing with a higher frequency [42]. Lower doses of 100 or 200 IU/day have also been tested, but several randomized trials support 400 IU/day as the best dose to prevent nutritional rickets with a minimum rate of side effects [42,46]. Both vitamin D2 and vitamin D3 have been used for the prophylaxis of nutritional rickets, with no significant differences in the rate of increase of 25(OH)D concentrations when given as a daily dose [47]. The use of vitamin D metabolites is not necessary for routine vitamin D treatment in infants, as they may increase the risk of hypercalcemia [48]. 

As calcium plays an important role in bone mineralization, optimal calcium treatment is required apart from vitamin D to prevent nutritional rickets [49]. Table 3 shows the recommended daily intake of calcium and vitamin D in children. Most calcium in a child’s diet comes from dairy products, and as the Western diet is usually rich in these products, children generally have sufficient calcium intake. However, some developing countries and other cultures traditionally consume few or no dairy products and have higher rates of nutritional rickets despite adequate levels of vitamin D [50,51].

Apart from vitamin D treatment, other risk factors for calcium or vitamin D deficiency should be avoided in childhood. Firstly, dietary diversification in infants should start no later than 6–6.5 months and include calcium-rich products to ensure adequate intake of these nutrients. Furthermore, special diets such as those that avoid cow’s milk and use soy or rice milk that are not specifically designed for infants (fortified), or vegetarian diets avoiding dairy products, may predispose infants to nutritional rickets [52,53].

There is no consensus on the need for chronic treatment or measurement of 25(OH) D serum levels in the at-risk groups described above (premature children, children with obesity, those with chronic diseases, or those taking medications that modify VDES metabolism) [21]. However, it is probably prudent and safe to study and treat calcifediol deficiency on a regular base in this population with a high risk of nutritional rickets.

### 6.2. Vitamin D for the Treatment of Nutritional Rickets

Nutritional rickets in children is defined as defective chondrocyte differentiation and mineralization of the growth plate and osteoid mineralization secondary to vitamin D deficiency and/or low calcium intake [21]. It is by far the most frequent cause of rickets in infancy. Although the administration of vitamin D in prophylactic doses to prevent nutritional rickets has dramatically reduced its incidence worldwide in the last few decades, it still is a matter of concern in populations with other additional risk factors and in some developing countries [54]. Children at risk for calcifediol deficiency, as stated above, are at a higher risk of developing nutritional rickets. Finally, children with nutritional rickets associated with vitamin D deficiency are at higher risk of bone fractures [55].

Various protocols have been employed for the treatment of nutritional rickets. High doses of vitamin D are used, ranging from 1500 IU/day to 300,000–600,000 IU as a starting bolus. The major concern about using higher doses is the possibility of producing severe hypercalcemia or secondary hypercalciuria with kidney calcifications (nephrocalcinosis/lithiases). However, multiple trials have proved higher doses to be safe with isolated cases of hypercalcemia or hypercalciuria associated with the use of high bolus doses at treatment initiation [56]. A recent Cochrane review has confirmed the absence of major side effects with the use of these higher doses, although the quality of evidence is stated as low or very low [45].

The usual recommended dose of vitamin D to treat nutritional rickets is 2000 IU/day, ranging from 1500 to 10,000 IU/day, for a minimum of 3 months [21,57]. These doses have been proven to be safe and effective, and normalize the typical biochemical findings of hyperparathyroidism, hypocalcemia, and hypophosphatemia. Low daily maintenance doses of 400 IU/day may be necessary after initial treatment, as these children have a higher risk of nutritional rickets afterward. Both vitamin D_3_ (cholecalciferol) and its metabolite (25(OH)D3; also called calcifediol or calcidiol) have been used effectively to treat nutritional rickets. Calcifediol results in a more rapid increase in serum levels of vitamin D, and lower doses are needed for the same effect compared with cholecalciferol. In addition, the intestinal absorption of calcifediol is higher and does not require hepatic hydroxylation, and this may be an advantage in certain pathologies [58].

Simultaneous administration of calcium (at a dose of 500–1000 mg per day) with vitamin D is usually recommended in children with nutritional rickets, as nutritional calcium deficiency is frequently associated. Various studies have shown that exclusive vitamin D treatment without calcium may normalize serum levels of 25(OH)D but not reverse clinical signs of rickets [21,59]. Moreover, calcium-deficient diets are very usual in children from developing countries, where the highest rates of nutritional rickets are reported.

### 6.3. Treatment of Genetic Forms of Rickets

As previously described, there are several forms of rickets of genetic origin, which commonly share an inability to maintain normal levels of active vitamin D despite appropriate treatment, either because of a defect in 25(OH)D to 1,25(OH)2D conversion, a defect in active vitamin D metabolites transport, or an excess in 1,25(OH)2D inactivation.

Patients with VDDR1A carry biallelic pathogenic variants in the *CYP27B1* gene. They characteristically present with very low plasma concentrations of 1,25(OH)2D despite treatment with high doses of calcifediol or cholecalciferol, reflecting an inability to activate vitamin D [60]. As a result, treatment with active forms of vitamin D is indicated: calcitriol or alfacalcidol. Calcium supplements may be required, as for the treatment of nutritional rickets, if clinical signs of rickets are present or dietary intake is not guaranteed.

Patients with VDDR1B carry biallelic pathogenic variants in the *CYP2R1* gene. This is a very rare condition, with few patients reported, which usually responds to treatment with calcifediol and calcium supplementation to treat nutritional rickets [12].

Patients with VDDR2A carry biallelic pathogenic variants in the *VDR* gene. The dysfunction of this receptor produces tissue resistance to 1,25(OH)2D, thus preventing its action. Depending on the severity of the receptor defect, patients may respond to high doses of active vitamin D and oral calcium supplementation to prevent hypocalcemia. Treatment is usually started with 1,25(OH)2D at a dose of 2 mcg/day with 1000 mg/day of oral calcium. However, some patients with very severe *VDR* dysfunction fail to respond to this treatment. If these doses fail to normalize biochemical findings, higher doses of active vitamin D (up to 60 mcg/day of calcitriol or alfacalcidol) may be used. In some patients, oral calcium at high doses (3 g/day) may not be well tolerated, and intravenous calcium infusions may be necessary to maintain normal calcemia and prevent progressive demineralization [61].

The last form of VDDR described (VDDR3) is secondary to activating mutations in *CYP450*, which increases the inactivation of VDES metabolites [15]. These patients present with the classic clinical findings of rickets and very low levels of both 25(OH)D and 1,25(OH)2D, and they apparently respond to treatment with very high doses of both metabolites.

## 7. Conclusions

Despite the variable definitions of calcifediol deficiency between the different scientific associations, nutritional rickets is a frequent worldwide pathology in childhood, especially in developing countries and children with other associated risk factors. Although infrequent, genetic forms of rickets should be considered in the diagnosis of a child with serum level alterations of VDES metabolites, as clinical and biochemical characteristics could be similar. Children without specific pathologies or additional risk factors should receive a sufficient amount of vitamin D and calcium to ensure adequate bone mineralization and prevent the development of nutritional rickets and its associated complications. Children with established rickets due to vitamin D deficiency, genetic alterations, or other chronic associated diseases should be managed adequately and treated with different forms of VDES metabolites and calcium to prevent other complications over time.

## Figures and Tables

**Table 1:** Calcifediol (25(OH)D) status definition according to different organisations and scientific societies (adapted from [5]); to convert to ng/mL, divide by 2.496.

	25(OH)D Deficiency nmol/L	25(OH)D Sufficiency nmol/L
Institute of Medicine	<30	>50
American Academy of Pediatrics	<50	
Endocrine Society	<25 (severe)	>75
European Society of Pediatric Endocrinology	<30 (severe)	>50
European Calcified Tissue Society	<20 (severe) <50 (deficiency)	>50

**Table 2:** Stages of severity in vitamin D deficiency rickets.

Stage	I	II	III
Serum calcium	↓	Normal	↓
Serum phosphate	N-↓	↓	↓
Serum alkaline phosphatase	↑	↑↑	↑↑↑
Calciuria	↓	↓	↓
Parathyroid hormone	↑	↑↑	↑↑↑
25(OH)D *	↓	↓	↓
1,25(OH)D	N-↓	N-↓	↓
Radiologic findings	None	Low bone density	Yes

N = normal; * 25(OH)D serum levels may be normal or even high if treatment with vitamin D has been started recently. ↓: mild decrease, N-↓: normal to low levels, ↑: mild increase, ↑↑: moderate increase, ↑↑↑: severe increase.

**Table 3:** Recommended dietary reference intakes (DRI) of calcium and vitamin D (modified from [49]).

Age	Calcium (mg/d)	Vitamin D (IU/d)
0–6 months	200	400
6–12 months	260	400
1–3 years	700	600
4–8 years	1000	600
9–13 years	1300	600
14–18 years	1300	600

## Data Availability

Not applicable.
